# The Diagnostic Odyssey of Patients with Chronic Neuropathic Pain—Expert Opinion of Greek Pain Specialists

**DOI:** 10.3390/clinpract13010015

**Published:** 2023-01-27

**Authors:** Persefoni Kritikou, Athina Vadalouca, Martina Rekatsina, Giustino Varrassi, Ioanna Siafaka

**Affiliations:** 1 Hellenic Society of Pain Management and Palliative Care (PARH.SY.A.), 11523 Athens, Greece; 2 Pain and Palliative Care Center, Athens Medical Center, Private Hospital, 11523 Athens, Greece; 3 Aretaieio Hospital, National and Kapodistrian University of Athens, 11528 Athens, Greece; 4 Paolo Procacci Foundation, 00193 Roma, Italy

**Keywords:** chronic pain, neuropathic pain, diagnostic odyssey, Greece

## Abstract

The diagnosis of chronic neuropathic pain requires a laborious process and can be a very long journey for the patients, one that can be characterized as an “odyssey.” Our aim was to describe the “diagnostic odyssey” associated with chronic neuropathic pain in the Greek context. Specialized clinicians working at dedicated chronic pain and palliative care centers were asked to participate in a survey regarding the diagnostic process in Greece. In total, 44 respondents provided information on the organization of their centers, the diagnostic process, and the perceived obstacles involved in the diagnosis of chronic neuropathic pain. Most respondents reported that their centers were not fully or efficiently organized and believed that additional specialized healthcare personnel should be employed. Raising public awareness about the existence of such centers was also considered key. The two main obstacles in reaching a diagnosis were the difficulty non-experts had in recognizing chronic neuropathic pain and the lack of acknowledgement that chronic neuropathic pain is a condition that needs to be addressed. When considering these responses in light of the extended socioeconomic burden associated with chronic neuropathic pain, efforts should be made to limit the “diagnostic odyssey” of chronic neuropathic pain in Greece. The aim of this study is to explore the experience of patients with chronic neuropathic pain in Greece from the viewpoint of pain specialists. A better organization of pain and palliative care centers, facilitation of communication with previously treating clinicians, increased personnel, utilization of a chronic pain registry, and guidelines development can aid in this venture. Keypoints: The diagnosis of chronic neuropathic pain in Greece is a laborious and time-consuming process that needs to be refined; Greek clinicians believe that their centers were not fully or efficiently organized and think that additional specialized healthcare personnel should be employed; Patient comorbidities and retards in visiting a clinic at the onset of symptoms delay the diagnosis of neuropathic pain and may complicate subsequent care; The diagnostic delay has been reported as three years between the onset of symptoms and seeking general medical help and another nine years before a referral to a pain specialist; Neuropathic pain is associated with patient distress and socioeconomic burdens, and diagnostic delays prolong the condition, may allow it to worsen, and utilize valuable healthcare resources without providing effective solutions.

## 1. Introduction

Chronic pain, which has been characterized as “invisible, subjective, and difficult to communicate” [1], may be the result of illness, injury, surgery, or cancer and can be categorized as a disease itself [2]. Neuropathic pain, resulting from lesions in the peripheral or central nervous system, most commonly affects people with diabetic peripheral neuropathy, postherpetic neuralgia (herpes zoster), and cancer [3]. Around 7% to 10% of adults have pain with neuropathic characteristics [4], and patient testimonials reveal a burden that is not fully appreciated by many healthcare professionals [5].

The mechanisms underlying neuropathic pain are not fully elucidated. In some cases, a lesion on a nerve or a somatosensory system disorder can trigger neuropathic pain symptoms [6]. Neuropathic pain is often perceived as qualitatively different from nociceptive pain because neuropathic pain may be associated with burning, tingling, “pins and needles,” and electrical-type sensations. Neuropathic pain can have a paroxysmal onset, be severe in intensity, or paradoxically be associated with a sense of numbness. Cellular and molecular signaling may be deranged, leading to maladaptive changes that can disturb ion channels and other mediators [6]. Neuropathic pain is associated with aberrant activity in ectopic nerves, central or peripheral sensitization, disrupted regulation of inhibitory modulation, and maladaptive microglial activation [6].

Despite the availability of several guidelines for the diagnosis and treatment of chronic neuropathic pain [7,8,9,10], a variety of factors contribute to the long diagnostic journey for patients trying to obtain effective chronic pain management. These factors include misdiagnosis by non-specialists, a lack of knowledge of symptomatic neuropathic pain treatment, and a general downplaying of patient self-reports of pain. [11,12]. It has been described in the literature that the challenges associated with the diagnosis and management of chronic pain can lead to conflicting opinions among healthcare providers, which may impede diagnosis altogether [13]. It has also been reported that these challenges can lead to up to three years of delay between the symptom onset/pain initiation and the patient’s first visit to a general practitioner and up to 12 years between the symptom onset/pain initiation and a referral to a specialized pain management and palliative care center [14], leading to a “diagnostic odyssey” for these patients.

A significant reason that may also lead to a delayed diagnosis is the development of different criteria throughout the years, such as the International Association for the Study of Pain (IASP) criteria for Complex Regional Pain Syndrome (CRPS). Underdiagnosis of this condition, for example, if the patient is not meeting all the criteria, might delay treatment of this type of neuropathic pain [15].

The diagnosis of chronic neuropathic pain requires a laborious process in order to identify its various components and decide on the most appropriate treatment [16,17]. Importantly, as it might coexist with other types of pain, its identification is fundamental [3]. An example of this “diagnostic odyssey” was related by a clinician who specializes in treating such patients. It was not until he was faced with chronic pain himself that he realized how much additional effort is required to fully understand chronic pain and the significant toll it takes on patients [13].

Published literature on patients with chronic neuropathic pain in Greece is limited. A study based on the Greek Neuropathic Pain Registry recently described the patient characteristics, treatment patterns, and clinical outcomes of 2334 patients with chronic neuropathic pain (from a total sample of 5980 patients with chronic pain) across several pain and palliative centers in Greece between 2016 and 2020 [16]. This research highlighted that the average time from pain initiation to referral to the pain clinics was 1.5 years [18]. This information has been confirmed in other countries as well [19]. Further outcomes from the Greek registry, focusing specifically on 168 patients treated in the Pain and Palliative Center of the Athens Medical Center, have also been presented [20]. Additional data on 120 patients treated in the University Hospital of Heraklion have been published as well, highlighting the significance of chronic neuropathic pain in Greece [21]. Nevertheless, the prevalence of chronic pain in Greece remains unknown, along with the “diagnostic odyssey” these patients endure.

Untreated chronic neuropathic pain can have devastating consequences for the individuals, such as depression, anxiety, and a concomitant reduced quality of life [18]. One study from the UK during the COVID-19 pandemic showed that after all scheduled spinal cord stimulation therapies for the management of chronic neuropathic pain were halted, pain, mental health, and the patient’s ability to self-manage pain deteriorated significantly [22].

Our aim was to describe the long and difficult pathway, or in other words, the “diagnostic odyssey,” of patients that suffer with chronic neuropathic pain in Greece, as reported by the specialists caring for them.

## 2. Materials and Methods

A structured questionnaire was sent to 51 Greek chronic pain and palliative care centers in order to collect information on the diagnostic process that follows entry into their pain and palliative care centers. These centers were selected based on their experience with treating neuropathic pain (based on the volume of patients seen yearly as well as their involvement in the Greek Neuropathic Pain Registry [19]) and represented specialized centers across Greece. In total, 44 of the 51 centers participated in the survey, representing approximately 75% of the 57 public chronic pain and palliative care centers currently established in Greece. One clinician from each center provided responses, for a total of 44 clinicians.

The survey collected information in five sections. First, the respondents provided the basic characteristics of their centers, including the definition used for chronic neuropathic pain, the process used to diagnose chronic neuropathic pain when the patient had not yet been diagnosed, and the proportion of chronic pain patients visiting the center due to chronic neuropathic pain specifically.

Second, the clinicians were asked about the organization of their centers, including the necessity of implementing specific activities to enhance their efficiency. The respondents rated, on a scale of 1 (not necessary at all) to 7 (absolutely necessary), the following predefined statements: employment of additional specialized healthcare professionals, more accurate documentation of the patients’ medical records, patient-physician communication that improved versus past visits, and raised public awareness regarding the existence and services provided by the pain management and palliative care centers.

Third, the survey collected information on the patients’ initial visit to the center and the diagnostic process (e.g., referral to these specialized centers from other physicians, prior confirmation of a chronic pain diagnosis, time between the symptom onset/pain initiation and the initial visit to the center). Respondents further provided their views on the complexity and difficulty in reaching a diagnosis, given the patient’s comorbidities, the perceived benefits of patients visiting the specialized centers earlier, and delays in diagnosis.

Finally, the survey collected information on the perceived satisfaction with the current diagnosis of chronic neuropathic pain in Greece and perceived obstacles in the diagnosis of chronic neuropathic pain (e.g., difficulty in recognizing chronic neuropathic pain by non-experts, lack of targeted diagnostic tools, lack of acknowledgement that chronic neuropathic pain is a condition that needs to be addressed, patient difficulty in fully describing their chronic neuropathic pain symptoms, and the lack of an appropriate healthcare structure in Greece).

Results are presented as frequencies (%) and means (standard deviation [SD]) for all descriptive analyses.

## 3. Results

Of the 44 specialized clinicians participating in this survey, 34 (77%) were anesthesiologists and pain specialists, while 10 (23%) were only anesthesiologists. Chronic neuropathic pain was mainly defined as “pain following injury/damage to the somatosensory system” (77%), and “pain lasting more than 3 months” (50%). All clinicians confirmed that the diagnosis of chronic neuropathic pain was based on the process outlined by the Hellenic Society of Pain Management and Palliative Care (PARHSYA) [19], and on average, 49% of patients seen in these pain and palliative care centers were diagnosed with chronic neuropathic pain (although large variations were observed across the centers, with some experts reporting that up to 80% of the patients visiting their centers had already been diagnosed with chronic neuropathic pain).

**Figure 1:**
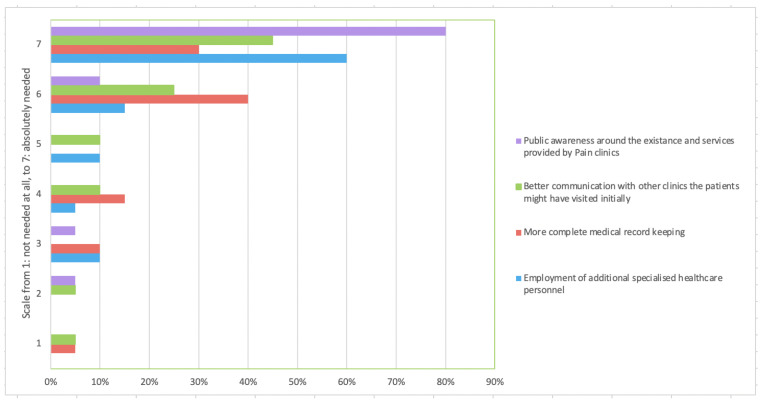
Actions needed to improve the efficiency of pain management and palliative care centers.

When asked about their center’s organization and efficiency, 40 (91%) respondents reported that their center was not fully or efficiently organized, and most of them felt that the following actions were “absolutely necessary” in order to improve the efficiency of their centers: employment of additional specialized healthcare professionals, more accurate documentation of the patients’ medical records, better communication with the physicians the patients might have visited in the past, and raising public awareness around the existence and services provided by the pain and palliative care centers (Figure 1).

As shown in Table 1, more than half of the patients (52%) came to the pain management and palliative care centers without any referral, while 25% of patients had received a diagnosis of chronic neuropathic pain at the time of their initial visit to these centers. The average time between symptom onset/pain initiation and the initial visit to these centers was 9.8 months (SD: 6.5).

When asked about the complexity of reaching a diagnosis given the patients’ comorbidities, on a Likert scale between 1 (not needed) and 7 (absolutely needed), most respondents ranked the difficulty as 4 (32%) or 5 (36%). Most importantly, the experts also reported that they felt the diagnosis could have been reached sooner if the patients had visited their center earlier. On a scale of 1 (do not agree at all) to 7 (totally agree), 23% of the respondents ranked this question as 6, while 73% of the respondents ranked this question as 7. The resulting delay in diagnosis was estimated to be 8.5 months on average (SD: 7.1).

When asked about their satisfaction with the current diagnosis process in Greece, most respondents replied that they were “relatively satisfied” (73%) or “not at all satisfied” (18%), as opposed to “very satisfied” (0%) and completely satisfied (9%).

**Figure 2:**
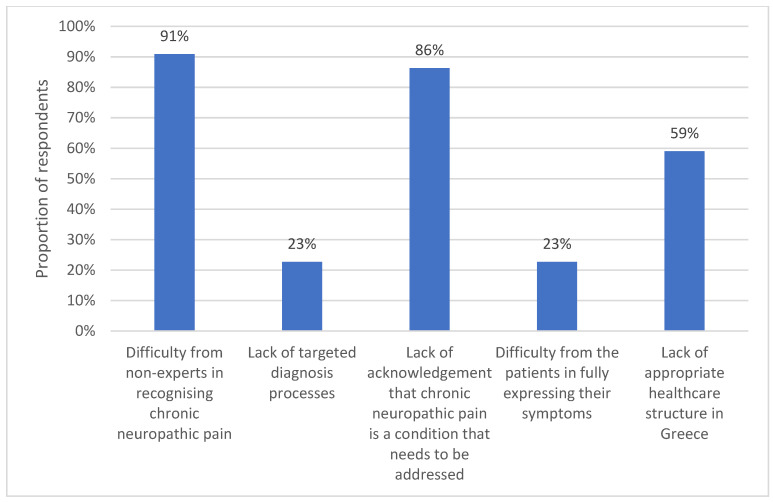
Reasons that hinder chronic neuropathic pain diagnosis.

The reasons that hinder the diagnosis of chronic neuropathic pain, as per the specialists, are mainly the difficulty non-experts have in recognizing chronic neuropathic pain and the lack of awareness that chronic neuropathic pain must be treated, followed by a lack of appropriate healthcare structure. A lack of targeted diagnostic processes and patient difficulty in expressing their symptoms seem to play a less significant role. The percentages are given in Figure 2.

## 4. Discussion

Based on the views of several key specialists in the management of chronic pain, the diagnosis of chronic neuropathic pain in Greece can indeed be characterized as an “odyssey.”

As many as 15 assessment tools are available to aid in the clinical diagnosis of neuropathic pain [23]. However, no “gold standard” of test has emerged. Many of these tests are based on patient self-reports of the characteristics of their painful symptoms and may require minimal or limited patient examination [24]. A challenge for neuropathic pain tests is the fact that neuropathic pain may differ by context, such as cancer versus non-cancer, or by condition, such as centralized back pain or trigeminal neuralgia, requiring different types of tests. Another important consideration is differentiating nociceptive from neuropathic pain, which guides treatment decisions. Patients and many clinicians may feel that pain is pain when, in reality, effective treatment depends on knowing the various underlying pain mechanisms. Patients may experience one type of pain or two or more types of pain concurrently [25]. Prominent among the tests used is the Leeds Assessment of Neuropathic Symptoms and Signs (LANSS), which requires both a patient interview and a physical examination. The Neuropathic Pain Questionnaire (NPQ) consists of 12 items for the patient to answer. The Douleur Neuropathique of Four Questions (DN4) has four questions and three tests that require a physical examination (touch, pin-prick, and light touch). PainDETECT is a patient survey with nine questions that include a body drawing for patients to use to describe pain locations [25].

Despite the recognition of the important role of primary care physicians in the identification of chronic neuropathic pain [9], according to the present survey, only roughly half of the patients visit specialized pain management and palliative care centers following a referral from other physicians, with just around a quarter of these patients having received a confirmed diagnosis of chronic neuropathic pain prior to their visit to these specialized centers. Considering that diagnosis in these specialized centers can be made relatively quickly (given the experience of the specialized clinicians), it poses the question of whether the “diagnostic odyssey” for chronic neuropathic pain patients could be shortened through the increased utilization of such centers in a more timely fashion. Given that the average time between symptom onset/pain initiation and the initial visit to these specialized centers was estimated to be more than 9 months, and the delayed diagnosis was estimated to be more than 8 months, the diagnostic process in Greece needs to be refined.

One potential explanation for the delayed diagnosis could be that the physicians treating the chronic neuropathic pain patients may lack knowledge of the condition. This could partly be attributed to the fact that chronic pain may be considered the result of a comorbidity, such as cancer or surgery, rather than a disease itself. Indeed, even though the International Classification of Diseases (ICD) 10th version included some codes for chronic pain, the shortcomings of this prior classification have been recognized, and as a result, the International Association for the Study of Pain (IASP), in collaboration with the World Health Organization (WHO), introduced a new classification of chronic pain in the newly released ICD 11th version [26,27]. Chronic neuropathic pain forms one of the seven main ICD-11 chronic pain categories and is further broken down into chronic central neuropathic pain (MG30.50) and chronic peripheral neuropathic pain (MG30.51) [27]. It remains to be seen how the implementation of these new codes might potentially shorten the “diagnostic odyssey” for chronic neuropathic pain patients.

Delays and hassles regarding the diagnosis and concomitant treatment of neuropathic pain have also been identified by German neurologists, who have a role in the management of chronic neuropathic pain. Among the reasons identified for this are the German diagnosis-related groups (DRG) reimbursement system, the insufficient academic posts, the lack of established pain centers, and a lack of focused pain education [28].

A comparative study conducted in Greece showed that “pain issue awareness” should be properly addressed in the medical curriculum so that future physicians, irrespective of their chosen specialty, are in a position to acknowledge the basic concepts of pain management. Almost all the students who attended the elective undergraduate course on chronic pain answered positively to the question that pain can be neuropathic in nature, but only two out of three students who had not attended the course were aware that neuropathic pain is an entity [29].

It should also be recognized that patients themselves may have attitudes toward pain and their illness that can delay the trajectory from symptom onset to diagnosis. Cultural attitudes may reflect how patients experience and interpret pain [30]. Religious faith has been a strong coping mechanism both for patients dealing with pain and for their family members and caregivers as they face the end of life [31]. In general, Greeks have a positive relationship with modern medicine, trust and confide in their physicians, and tend to be willing to accurately and fully describe their symptoms, particularly pain and discomfort. However, older individuals in Greece, as is the case in other parts of the world, may be more skeptical about modern healthcare and mistrust pain medications. All over the world, cancer can be a fear-inspiring diagnosis that may make patients reluctant to describe symptoms. Pain in a cancer patient may be feared as outright proof that the disease is getting worse, so there may be hesitancy to bring this to the attention of the physician [32]. Furthermore, cancer patients are sometimes reticent to discuss pain with their oncologists, for fear that focusing on their pain treatments might distract them from the more urgent work of getting rid of the cancer [32].

The terminology of “diagnostic odyssey” is commonly used in the space of rare diseases, where the inherent challenges of these diseases have resulted in specific actions, such as the ‘Ending the Diagnostic Odyssey Act of 2021′ in the United States (“To enable States to better provide access to whole genome sequencing clinical services for certain undiagnosed children under the Medicaid program, and for other purposes”) [33,34]. If such efforts are made for small patient populations, one could advocate that even more action should be taken to relieve the “diagnostic odyssey” associated with chronic neuropathic pain [35].

An important step to help enhance the timely diagnosis of chronic neuropathic pain, as reported by the clinicians in this survey, is the better structural organization of the health care system, such that the physicians initially treating patients who might be experiencing chronic neuropathic pain (e.g., primary care physicians) are able to recognize and refer these patients to specialized chronic pain and palliative care centers [36]. Pain patients, regardless of pain etiology, may experience diminished quality of life and functional deficits [37]. The burden that neuropathic pain places on the healthcare system and society at large is vast and likely underappreciated [38,39]

Neurologists are one of the main specialties that will significantly aid in reducing this burden. A focused education program is crucial as it is known that neurodegenerative [39] and neurologic disorders [16] can be a cause of neuropathic pain. Early diagnosis and treatment of the pain can significantly restore function.

It is also key to maintain an accurate repository of the patients’ medical records in order to promote knowledge of this condition and facilitate the effective management of these patients. An effort is already being made by the Greek Neuropathic Pain Registry, which has recorded information, including the diagnostic process, for more than 2334 patients with chronic neuropathic pain in Greece [19]. The main published results from this registry have highlighted the fact that patients with neuropathic pain in Greece are underdiagnosed outside of specialized pain management and palliative care centers [19]. As such, the establishment of additional specialized pain management and palliative care centers might be warranted, as they are considered the gold standard for treating chronic pain [26].

Chronic pain, and neuropathic pain specifically, has also been associated with a great socioeconomic burden in Greece, which can be comparable to other parts of Europe [27,33]. According to a study conducted in France, Germany, Italy, Spain, and the UK, the total annual direct healthcare costs per patient ranged from €1939 in Italy to €3131 in Spain; the annual professional caregiver costs ranged from €393 in France to €1131 in Spain (not accounting for informal caregivers); and the sick leave costs ranged from €5492 in the UK to €7098 in France, for a total cost (including direct and indirect costs for neuropathic pain) of up to €14,446 per patient [33]. A more efficient diagnosis process could help limit this economic burden by optimally addressing the needs of these patients and their caregivers with minimal delays in the care provided.

The burden of neuropathic pain hits certain patient populations particularly hard. For example, cancer outpatients in palliative care have a high burden of neuropathic pain; a study of 261 patients in India found 54% suffered from pain with a predominant neuropathic component [40]. A review from Japan reported a high burden for neuropathic back pain that affected not only physical function but social and mental well-being [41]. Anxiety, depression, and other mental health conditions add to the burden associated with neuropathic pain [42]. Few studies have explored the qualitative differences in pain burden between nociceptive and neuropathic pain patients. In an intriguing study from Brazil, 49 women living with HIV and chronic pain were divided into three groups: those with nociceptive pain, those with neuropathic pain, and the control group. Those with neuropathic pain had significantly more frequent pain, depression, and lower resilience when compared to control patients, and reported that pain interfered with their activities [43]. This study underscores what some clinicians have learned anecdotally from real-world clinical practice, namely that neuropathic pain is not only a different type of pain mechanism, but it can also cause significantly more severe symptoms and lifestyle effects.

One challenge to more timely diagnosis of neuropathic pain in any context is the fact that patients may be unaware that there are different types of pain. A patient may report pain to a physician, be inadequately treated for pain, and attribute it to weak medication rather than a wrong diagnosis of the underlying pain mechanism. A study from France reported that when patients were provided by their pharmacists with educational materials about neuropathic pain conditions, this improved their compliance, was generally received favorably, and might reduce inappropriate use of over-the-counter pain relievers [44]. Educational interventions can also help patients better learn to manage their symptoms and develop coping skills. In a study of 109 patients with spinal cord injury and neuropathic pain, 72.5% were taking two or more pain medications at the outset of the intervention; this proportion decreased to 33.0% at the conclusion of the intervention. The goal of this intervention was to explain to patients the nature and origin of neuropathic pain, management techniques, and the limited role of pain medication to manage this condition [45]. Thus, patient education can be divided into three main components: education and awareness of the existence of neuropathic pain as distinct from other types of pain, understanding of how to use medications properly in its treatment, and the role of other therapeutic techniques to help control neuropathic pain. At present, all are neglected. This corresponds with a need for clinician education to better understand the differences between nociceptive and neuropathic pain, diagnostic strategies to recognize neuropathic pain, and appropriate, effective treatments.

Certain limitations of this analysis should be recognized. First, the survey was based on a sample population of chronic pain experts in Greece. As such, these results might not be generalizable to the whole of Greece or Europe, even though these experts are very experienced with this issue. In addition, these results only reflect the perspectives of the clinicians specializing in chronic pain, while the perspectives of other healthcare professionals involved in the diagnosis (such as the primary care physicians who might be consulted by the patients initially) were not captured here. Furthermore, a key part of the “diagnostic odyssey” of chronic neuropathic pain, which was not included in this analysis, is the patient experience, which, according to a study in Kuwait, included very long waiting times to see a specialist, a lack of dedicated care for such patients, inefficiencies in the patients’ recording of medical files, and a general perception of unsatisfactory care [46]. As the purpose of the current research was to initiate a discussion on the “diagnostic odyssey” associated with chronic neuropathic pain in Greece, further research is needed to substantiate its impact on the whole Greek population. Additionally, the questionnaire used in our research has not been validated. However, to our knowledge, there has been no prior research using a similar questionnaire. Even though previous studies have used similar methodologies (i.e., surveying clinicians), they predominantly focused on the challenges associated with the management of chronic pain rather than diagnosing neuropathic pain symptoms [47,48]. This further highlights the fact that the diagnosis of chronic pain requires more dedicated efforts.

Another limitation to our survey is inherent in all surveys: response bias. People who agree to take surveys are fundamentally different from those who refuse to participate. Finally, we treated all types of neuropathic pain as the same, although there are many different types of neuropathic pain. For example, chemotherapy-induced peripheral neuropathy differs from neuropathic pain following failed back syndrome, which differs from pain in cluster headaches or in HIV. The full ramifications of these different types of neuropathic pain are not well studied, and most trials involving neuropathic pain patients use neuropathic pain specific to a disease, condition, or context. It is also unclear if neuropathic pain in a palliative patient differs from neuropathic pain in others. For that reason, further studies involving more specific populations or capturing the distinctions among populations would be most interesting.

We encourage future studies that use validated tools and are addressed not only to clinicians but also to patients, who are the actual sufferers. Additionally, by expanding the search to general practitioners or neurologists, a clearer picture might be identified.

## 5. Conclusions

As indicated by the views of the chronic pain specialists included in this survey, the diagnostic process for chronic neuropathic pain in Greece is long and complicated. The main obstacles are the lack of knowledge among non-experts regarding neuropathic pain (identification and significance of treatment), the lack of an appropriate organizational structure in specialized pain management centers, and certain difficulties faced by the patients. To efficiently identify and manage chronic neuropathic pain, the employment of additional specialized healthcare professionals, the existence of more accurate documentation of the patients’ medical records, direct communication with previous treating physicians, and raising public awareness about the existence and services provided by the pain and palliative care centers are crucial. The usage of tools, such as the Neuropathic Pain Registry of the Hellenic Society of Pain Management and Palliative Care (PARHSYA), as well as guidelines development, can further facilitate the above need. A concentrated effort by Greek authorities could possibly resolve this major problem and limit the “diagnostic odyssey” for patients with chronic neuropathic pain.

## Figures and Tables

**Table 1:** Initial Visit and Diagnosis Process.

Initial Visit		
Process Leading to the Initial Visit (%)		
	Patients referred to the centre by other healthcare professionals	48%
	Patients coming directly to the centre without any referral	52%
Has a diagnosis of chronic neuropathic pain been reached at the time of initial visit? (%)		
	Yes	25%
	No	75%
What is the average time between pain initiation and initial visit to the center? (months)		
	Mean (SD)	9.8 (6.5)
Diagnostic Process		
How complex is diagnosis, given other comorbidities? (%)		
	Scale from 1 (not complicated at all) to 7 (very complicated)	
	1	5%
	2	14%
	3	5%
	4	32%
	5	36%
	6	5%
	7	5%
Do you believe that the diagnosis could have been reached earlier, if the patient had visited the center earlier? (%)		
	Scale from 1 (not at all) to 7 (absolutely yes)	
	1	0%
	2	0%
	3	0%
	4	5%
	5	0%
	6	23%
	7	73%
What is the average time of diagnosis delay? (months)		
	Mean (SD)	8.5 (7.1)

SD: standard deviation.

## Data Availability

The data used for these analyses are available by the Corresponding Author, on reasonable request.
